# A Novel Indolizine Derivative Induces Apoptosis Through the Mitochondria p53 Pathway in HepG2 Cells

**DOI:** 10.3389/fphar.2019.00762

**Published:** 2019-07-10

**Authors:** Yushuang Liu, Enxian Shao, Zhiyang Zhang, Daji Yang, Guanting Li, Hua Cao, Hongliang Huang

**Affiliations:** ^1^School of Biosciences & Biopharmaceutics and Center for Bioresources & Drug Discovery, Guangdong Pharmaceutical University, Guangzhou, China; ^2^School of Pharmacy, Guangdong Pharmaceutical University, Guangzhou, China; ^3^School of Chemistry and Chemical Engineering, Guangdong Pharmaceutical University, Zhongshan, China; ^4^Guangzhou Key Laboratory of Construction and Application of New Drug Screening Model Systems, Guangdong Pharmaceutical University, Guangzhou, China

**Keywords:** indolizine derivatives, HepG2 cells, apoptosis, mitochondrion, p53

## Abstract

Indolizine derivatives are a class of compounds with excellent biological activity. In this study, a series of indolizine derivatives, compound 1 (C1), compound 2 (C2), compound 3 (C3), and compound 4 (C4), were synthesized. 3-(4,5-dimethylthiazole)-2,5-diphenyltetraazolium bromide (MTT) assay was used to evaluate their cytotoxicity against HepG2 (p53-wild), A549, and HeLa cell lines. HepG2 cells apoptosis induced by C3 was determined using Hoechst staining and acridine orange/ethidium bromide staining. Cells’ apoptotic ratio was measured by Annexin V–FITC/PI double staining. Changes in mitochondrial membrane potential and intracellular reactive oxygen species (ROS) in HepG2 cells after C3 treatment were determined. Immunofluorescence staining and Western blot analysis were carried out to detect p53 levels and analyze the apoptosis-associated proteins, respectively. Moreover, the cytotoxic activity of C3 was examined in two other hepatocellular carcinoma (HCC) cell lines with different p53 status including Huh-7 cells (p53-mutant) and Hep3B cells (p53-null). The results indicated that C3 showed stronger inhibition towards HepG2 cells than other cell lines. Fluorescent staining and flow cytometry analysis confirmed that C3 induced apoptosis of HepG2 cells. C3 could also increase intracellular ROS and cause a decrease in the mitochondrial membrane potential. C3 promoted p53 activation and increased p53 accumulation in nuclei. The expression of p53 and Bax was increased with the down-regulation of Bcl-2, which promoted the release of cytochrome c and caspase-3 activation. Collectively, the study demonstrated that C3 caused HepG2 cell apoptosis *via* the mitochondria p53 pathway. These results inspired us to further develop indolizine derivatives as potential potent inhibitors against liver cancer.

## Introduction

**Graphical Abstract:**
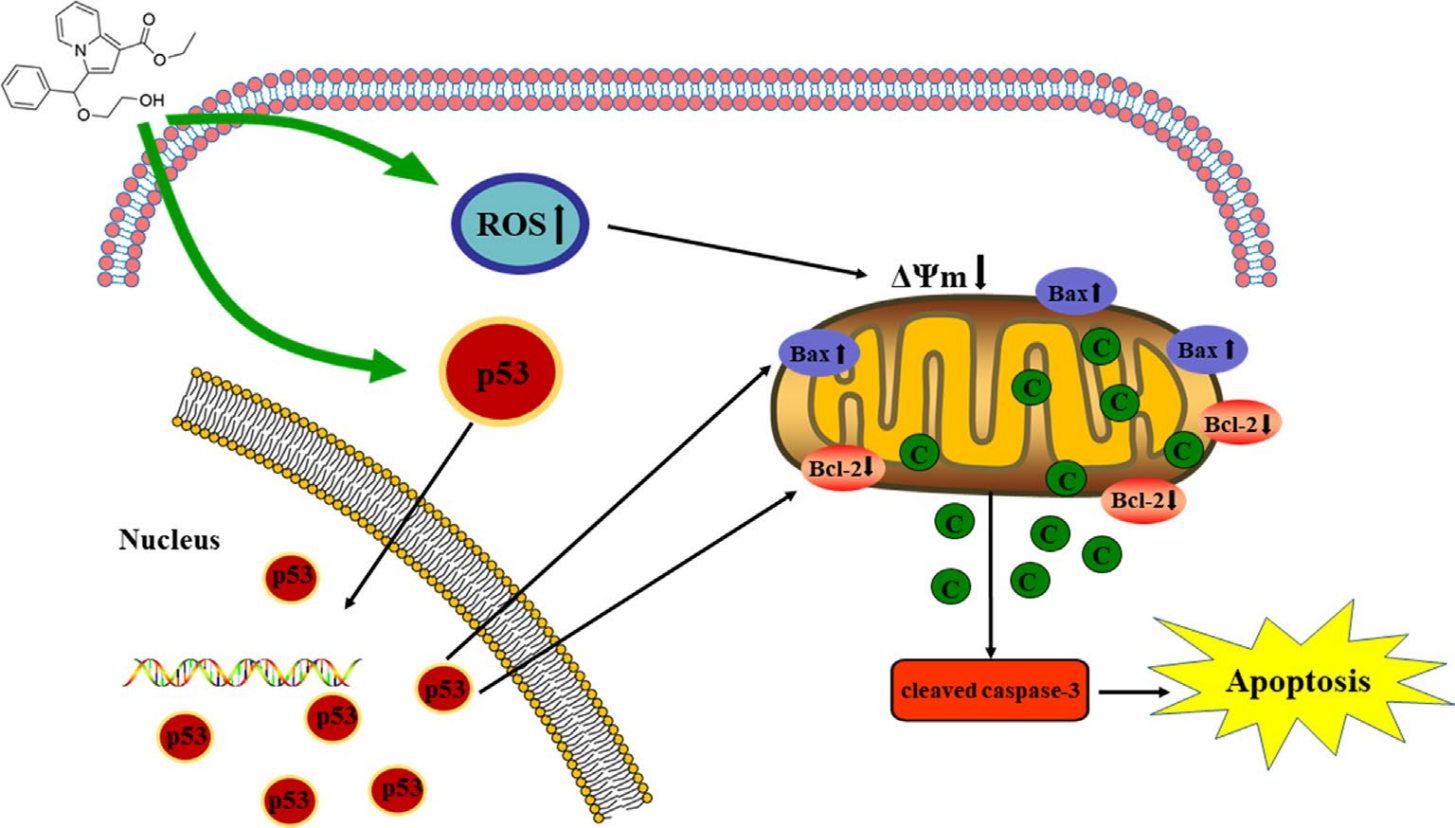
The proposed mechanisms of C3 inducing apoptosis in HepG2.

Indolizine, an important class of heterocycles, has attracted critical attention of chemists for its application value. They are found in a large number of natural products such as (±)-homocrepidine A (Hu et al., 2015), tashiromine (Marsden and McElhinney, 2008), swainsonine (Lee et al., 2013), Pandalisines A and B (Cheng et al., 2015), Flueggedine (Zhao et al., 2013), and so on. Recent studies have indicated that indolizines exhibit a broad range of biological activities including anti-herpesvirus (De Bolle et al., 2004), cyclooxygenase and lipoxygenase inhibition (Shrivastava et al., 2017), inhibition of *Mycobacterium tuberculosis* (Gundersen et al., 2007), and Ach receptor agonist (Xue et al., 2016).

**Figure 1:**
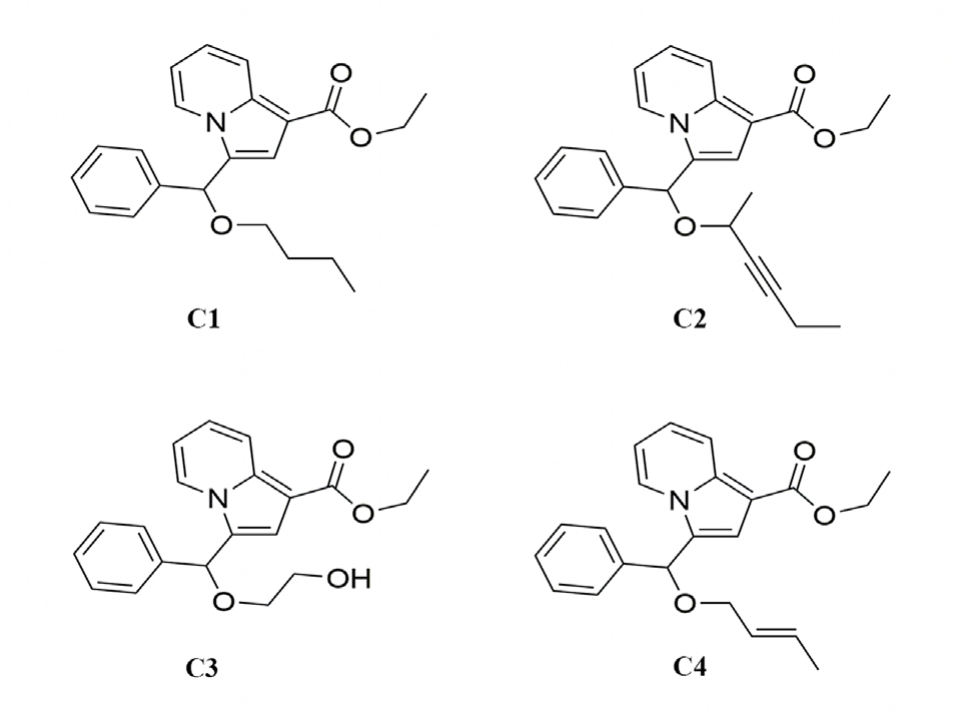
The chemical structure of indolizine derivatives.

There are some reports that indolizine derivatives have anti-tumor activity. It has been found that indolizine derivatives possessed anti-proliferative effect on melanoma cell lines MDA-MB-435 (Ghinet et al., 2015). Dumea et al. designed and synthesized indolizine derivatives that targeted the protein farnesyl transferase (Dumea et al., 2014). In addition, a series of indolizine derivatives synthesized by Shen et al. could inhibit the proliferation of HepG2 cells and showed significant epidermal growth factor receptor (EGFR) kinase inhibitory activity (Shen et al., 2010). Not only that, some research found that indolizine derivatives could also induce apoptosis in A549 cells (Lv et al., 2012; Moon et al., 2016). In this study, four novel indolizine derivatives were synthesized (**Figure 1**) in a brand-new way. However, their anti-tumor activities and the underlying mechanisms have not been fully elucidated. Therefore, the aim of this study was to investigate the optimal activity of four new indolizine derivatives synthesized on a variety of cancer cells and to explore the related mechanisms.

## Materials and Methods

### Materials

All chemical agents were purchased from Sigma-Aldrich (St. Louis, MO, USA). The cell lines of HepG2 (p53-wild), A549, and HeLa were obtained from the American Type Culture Collection. Huh-7 (p53-mutant) and Hep3B (p53-null) were acquired from the Cell Bank of Type Culture Collection of the Chinese Academy of Science (Shanghai, China). Dulbecco’s Modified Eagle’s Medium (DMEM), Roswell Park Memorial Institute (RPMI) 1640 Medium, and fetal bovine serum (FBS) were products of Thermo Fisher Scientific (Waltham, MA, USA). Primary antibodies against Bax, Bcl-2, caspase-3, cyto c, β-actin, and p53 were from Cell Signaling Technology (Danvers, MA, USA). The secondary antibodies were purchased from Beyotime Biotechnology (Shanghai, China). Pifithrin-α (PFTα) was purchased from Selleck Co. Ltd.

### Cell Culture

HepG2, Huh-7, Hep3B, and HeLa cells were cultured in Dulbecco’s Modified Eagle’s Medium (DMEM). A549 cells were cultured in RPMI 1640 Medium. All cell lines were supplemented with 10% FBS and 1% penicillin/streptomycin and maintained at 37°C in a humidified atmosphere with 5% CO_2_.

### Synthesis and Characterization

#### Synthesis of C1

A 25-ml Schlenk tube was charged with a stirring bar, and ethyl 2-(pyridin-2-yl)acetate (**1a**, 49.6 mg or 165.2 mg), phenyl-propynal (**2a**,39.0 mg or 130.2 mg), n-butyl alcohol (**3c**, 222.3 mg or 741.1 mg), PivOH (6.1 mg or 20.4 mg), and 5A MS (150.0 mg or 450.0 mg) were added. The reaction was allowed to stir at room temperature under Ar atmosphere for 2 h. The crude product was separated by column chromatography (eluted with petroleum ether:ethyl acetate:triethylamine = 40:5:1) to give a pure sample of **C1** in a 72% or 70% yield (75.9 mg or 246.0 mg), respectively. IR (KBr): 1747, 1654, 1355, 1148, 548 cm^−1^. ^1^H NMR (400 MHz, CDCl_3_) δ 8.21 (d, *J* = 9.2 Hz, 1H), 8.07 (d, *J* = 7.2 Hz, 1H), 7.42–7.30 (m, 5H), 7.09–7.05 (m, 1H), 6.99 (s, 1H), 6.68–6.65 (m, 1H), 5.68 (s, 1H), 4.33 (q, *J* = 7.2 Hz, 2H), 3.54–3.43 (m, 2H), 1.65–1.58 (m, 2H), 1.43–1.36 (m, 5H), 0.88 (*t*, *J* = 7.2 Hz, 3H). ^13^C NMR (100 MHz, CDCl_3_) δ 165.0, 138.7, 137.0, 128.5, 127.9, 126.8, 125.1, 124.2, 122.5, 119.8, 117.6, 112.1, 102.8, 76.7, 68.7, 59.4, 31.8, 19.4, 14.6, 13.8.

#### Synthesis of C2

A 25-ml Schlenk tube was charged with a stirring bar, and ethyl 2-(pyridin-2-yl)acetate (**1a**, 49.6 mg or 165.2 mg), phenyl-propynal (**2a**, 39.0 mg or 130.2 mg), hex-3-yn-2-ol (**3n**, 294.4 mg or 981.4 mg), PivOH (6.1 mg or 20.4 mg), and 5A MS (150.0 mg or 450.0 mg) were added. The reaction was allowed to stir at room temperature under Ar atmosphere for 2 h. The crude product was separated by column chromatography (eluted with petroleum ether:ethyl acetate:triethylamine = 40: 5: 1) to give a pure sample of **C2** in a 68% or 66% yield (76.6 mg or 247.8 mg), respectively. IR (KBr): 1733, 1659, 1328, 1166, 535 cm^−1^. ^1^H NMR (400 MHz, CDCl_3_) δ 8.62 (d, *J* = 7.2 Hz, 1H), 8.19 (d, *J* = 9.2 Hz, 1H), 7.50–7.40 (m, 4H), 7.14–7.09 (m, 1H), 6.80–6.77 (m, 1H), 6.59 (s, 1H), 6.09 (s, 1H), 4.28 (q, *J* = 7.2 Hz, 2H), 4.16–4.11 (m, 1H), 2.34–2.28 (m, 2H), 1.42 (d, *J* = 6.8 Hz, 3H), 1.33 (s, 3H), 1.23 (s, 3H).^13^C NMR (100 MHz, CDCl_3_) δ 165.0, 137.8, 136.9, 128.7, 128.4, 127.8, 125.1, 122.7, 119.8, 117.6, 112.2, 102.9, 87.9, 79.2, 73.6, 63.0, 59.4, 29.7, 22.5, 14.6, 14.0, 12.4.

#### Synthesis of C3

A 25-ml Schlenk tube was charged with a stirring bar, and ethyl 2-(pyridin-2-yl)acetate (**1a**, 49.6 mg or 165.2 mg), phenyl-propynal (**2a**, 39.0 mg or 130.2 mg), ethane-1,2-diol (**3f**, 186.2 mg or 620.7 mg), PivOH (6.1 mg or 20.4 mg), and 5A MS (150.0 mg or 450.0 mg) were added. The reaction was allowed to stir at room temperature under Ar atmosphere for 2 h. The crude product was separated by column chromatography (eluted with petroleum ether:ethyl acetate:triethylamine = 40:5:1) to give a pure sample of **C3** in a 65% or 62% yield (66.2 mg or 210.4 mg), respectively. IR (KBr): 3508, 1657, 1603, 1359, 1149 cm^−1^. ^1^H NMR (400 MHz, CDCl_3_) δ 8.18 (d, *J* = 9.2 Hz, 1H), 8.14 (d, *J* = 6.8 Hz, 1H), 7.41–7.30 (m, 5H), 7.07–7.03 (m, 1H), 6.90 (s, 1H), 6.68 (t, *J* = 6.8 Hz, 1H), 5.74 (s, 1H), 4.31 (q, *J* = 7.2 Hz, 2H), 3.80–3.71 (m, 2H), 3.63-3.53 (m, 2H), 3.09 (s, 1H), 1.35 (t, *J* = 7.2 Hz, 3H). ^13^C NMR (100 MHz, CDCl_3_) δ 164.9, 137.9, 137.0, 128.5, 128.1, 126.9, 124.9, 124.0, 122.7, 119.8, 117.6, 112.4, 102.8, 77.0, 67.0, 61.8, 59.4, 14.5.

#### Synthesis of C4

A 25-ml Schlenk tube was charged with a stirring bar, and ethyl 2-(pyridin-2-yl)acetate (**1a**, 49.6 mg or 165.2 mg), phenyl-propynal (**2a**, 39.0 mg or 130.2 mg), (*E*)-but-2-en-1-ol (**3j**, 216.3 mg or 721.1 mg), PivOH (6.1 mg or 20.4 mg), and 5A MS (150.0 mg or 450.0 mg) were added. The reaction was allowed to stir at room temperature under Ar atmosphere for 2 h. The crude product was separated by column chromatography (eluted with petroleum ether:ethyl acetate:triethylamine = 40:5:1) to give a pure sample of **C4** in a 72% or 67% yield (75.5 mg or 234.1 mg), respectively. IR (KBr): 2,954, 1,689, 1,613, 1,358, 1,148 cm^−1^. ^1^H NMR (400 MHz, CDCl_3_) δ 8.20 (d, *J* = 9.2 Hz,1H), 8.08 (d, *J* = 7.2 Hz, 1H), 7.42–7.32 (m, 5H), 7.09–7.05 (m, 1H), 7.00 (s, 1H), 6.68–6.65 (m, 1H), 5.77 (s, 1H), 5.74–5.61 (m, 2H), 4.34 (q, *J* = 7.2 Hz, 2H), 3.95 (d, *J* = 4.4 Hz, 2H), 1.72 (d, *J* = 5.6 Hz, 3H), 1.38 (t, *J* = 7.2 Hz, 3H). ^13^C NMR (100 MHz, CDCl_3_) δ 165.0, 138.4, 137.0, 130.4, 128.5, 127.9, 126.9, 126.8, 125.1, 123.9, 122.5, 119.8, 117.8, 112.2, 102.9, 75.3, 69.1, 59.4, 17.8, 14.6.

### Cell Viability Assay

The cytotoxicity effect of indolizine derivatives was determined by MTT assay. In brief, the cell lines (HepG2, A549, HeLa, Huh-7, and Hep3B) were seeded in 96-well plates at a density of 1 × 10^5^ cells/well. After incubation for 24 h, the cells were incubated with different concentrations of indolizine derivatives for 24 or 48 h. Then, the culture medium was discarded and we added 100 μl of fresh culture medium containing 10 μl MTT (5 mg/ml) per well and further incubated for 4 h. The supernatant was then removed, and the formazan was dissolved in 100 µl of dimethyl sulfoxide (DMSO) for 10 min. The absorbance at 570 nm was measured by an enzyme-labeled instrument (Thermo Labsystems, USA).

### Nuclei Morphology Detection by Hoechst 33258

Hoechst 33258 staining was performed by following the protocol of Hoechst Staining Kit (Beyotime Biotechnology, China). The cells were seeded in 12-well plates and cultured for 24 h. The cells were incubated with or without different concentrations of C3 (20 and 40 µg/ml) for 24 h. Then, the cells were washed once with PBS. Cells were fixed with 4% paraformaldehyde for 10 min at room temperature and washed twice with PBS. The cells were stained with Hoechst 33258 staining solution for 5 min; then, cells were washed twice with PBS. The morphology of cell nuclei was examined under a fluorescence microscope (Zeiss, Germany).

### Acridine Orange/Ethidium Bromide (AO/EB) Staining

The cells were incubated in the absence or presence of C3 at different concentrations (20 and 40 µg/ml) at 37°C and 5% CO_2_ for 24 h. After treatment, the medium was removed and the cells were washed with ice-cold PBS. Cells were stained with AO/EB solution for 10 min (AO: 100 µg/ml, EB: 100 µg/ml). The cells were observed and imaged with a fluorescence microscope.

### Apoptosis Assay by Flow Cytometry

Apoptotic cell death was detected using Annexin V–FITC Apoptosis Detection Kit (Beyotime Biotechnology, China) in accordance with the manufacturer’s instructions. The cells were harvested and washed with ice-cold PBS. Subsequently, the cells were resuspended in 195 µl of binding buffer and stained with 5 µl of Annexin V–FITC and 10 µl of propidium iodide by incubation in the dark at room temperature for 15 min. The prepared cellular samples were immediately performed using a CytoFLEX flow cytometer (Beckman Coulter) and analyzed by the CytExpert software program (Beckman Coulter).

### Determination of Mitochondrial Membrane Potential (MMP, ΔΨm)

The alteration of ΔΨm in HepG2 cells was detected using an MMP assay kit with JC-1 (Beyotime Biotechnology, China). The cells were incubated with or without C3 (20 and 40 µg/ml) for 24 h. The cells were collected and stained with 5 μg/ml JC-1 at 37°C for 30 min in the dark. Cells were then washed twice with JC-1 buffer (1×). The samples were kept on ice and measured by a flow cytometer immediately.

### Measurement of Reactive Oxygen Species (ROS)

Measurement of intracellular changes in ROS generation was performed using an ROS assay kit (Beyotime Biotechnology, China). The cells were incubated with C3 (20 and 40 µg/ml) for 24 h and the untreated cells were maintained as the control. The cells were stained with 10 μM DCFH-DA (2′,7′-Dichlorodihydrofluorescein diacetate) at 37°C for 30 min followed by washing with PBS. Finally, the fluorescent signals were detected immediately with flow cytometer.

### Immunofluorescence Staining

The cells incubated with or without C3 (20 and 40 µg/ml) were fixed with 4% formaldehyde for 15 min. Then, the cells were permeabilized in 0.5% Triton X-100 in PBS for 15 min and blocked with 5% BSA for 30 min. The cells were subsequently incubated with primary antibody for p53 (Cell Signaling Technology) at 1:2,000 dilution overnight for 24 h at 4°C. After that, the cells were rinsed with PBS and simultaneously incubated with Alexa Fluor 488 anti-rabbit secondary antibodies (Beyotime Biotechnology, China) at 1:500 dilution for 1 h at room temperature. Nuclei were stained with 4’,6-diamidino-2-phenylindole (DAPI, Beyotime Biotechnology, China) for 5 min in the dark. Fluorescent signals were imaged under a fluorescence microscope.

### Western Blot Analysis

After 24-h incubation with C3, the cells were washed twice with ice-cold PBS and lysed in cell lysis buffer for Western and IP (Beyotime Biotechnology, China) supplemented with 1 mM phenylmethyl sulfonyl fluoride (PMSF, Beyotime Biotechnology, China). Protein concentrations were determined using BCA protein assay kit (Beyotime Biotechnology, China). Equal amounts of protein were separated on 12% SDS-PAGE gel and transferred to polyvinylidene fluoride (PVDF) membranes (Millipore, USA). The membranes were then blocked with 5% non-fat dry milk in tris-buffered saline tween20 (TBST) for 2 h at room temperature and followed by TBST washing three times, each lasting for 15 min. The membranes were incubated with primary antibodies for Bcl-2 (1:3,000), Bax (1:3,000), p53 (1:2,000), cleaved caspase 3 (1:2,000), cyto c (1:2,000), and β-actin (1:3,000) at 4°C overnight. Subsequently, the membranes were washed with TBST followed by incubation with the secondary antibody (1:3,000) at room temperature for 1 h. Then, the membranes were washed three times with TBST. Finally, the blots were developed with enhanced chemiluminescence substrate solution kit (ECL, Biosharp, China) and captured using Tanon 5200 Imaging Analysis System (Tanon, China). Image J software was used to analyze pictures.

###  Statistical Analysis

All the experiments were performed independently three times. The data were expressed as mean ± S.D. Statistical analysis was performed with PRISM 7.0 (GraphPad software). The differences of groups were assessed by the Student’s *t* test. Differences were considered significant at *P* < 0.05.

## Results

### Chemistry

**Scheme 1:**
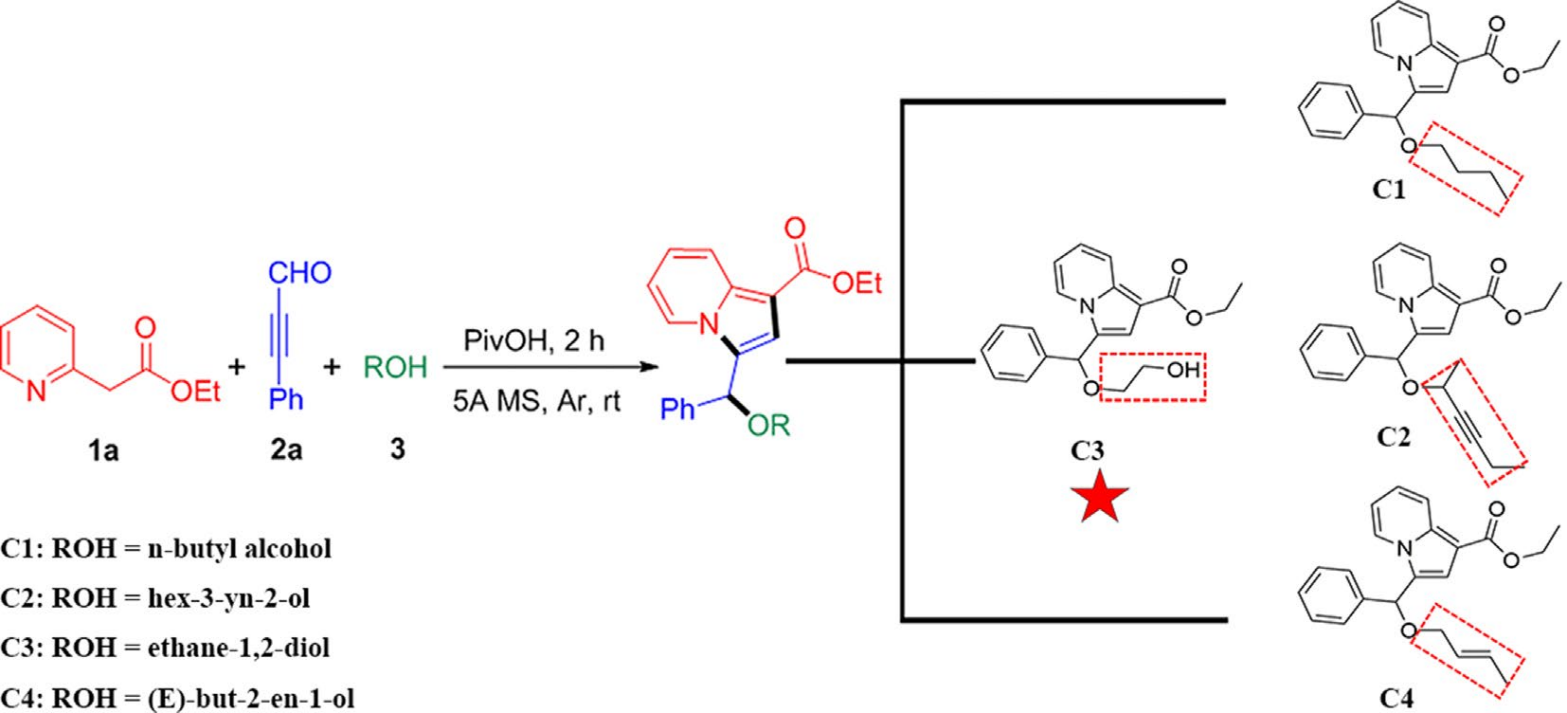
Synthetic route for indolizine derivatives (**C1, C2, C3, and C4**).

Recently, we have reported a novel, straightforward, and efficient strategy for the synthesis of functionalized indolizines in good yields under solvent- and metal-free conditions (Yang et al., 2018). We synthesized four important indolizine derivatives using this method in **Scheme 1**. A 25-ml Schlenk tube was charged with a stirring bar, and ethyl 2-(pyridin-2-yl)acetate (**1a**, 49.6 mg or 165.2 mg), phenyl-propynal (**2a**, 39.0 mg or 130.2 mg), reactants 3 [**n-butyl alcohol**, 222.3 mg or 741.1 mg; **hex-3-yn-2-ol**, 294.4 mg or 981.4 mg; **ethane-1,2-diol**, 186.2 mg or 620.7 mg; **(*E*)-but-2-en-1-ol**, 216.3 mg or 721.1 mg], PivOH (6.1 mg or 20.4 mg), and 5A MS (150.0 mg or 450.0 mg) were added. The reaction was allowed to stir at room temperature under Ar atmosphere for 2 h. The crude product was separated by column chromatography (eluted with petroleum ether:ethyl acetate:triethylamine = 40:5:1) to give a pure sample of **C1** in a 72% or 70% yield (75.9 mg or 246.0 mg), **C2** in a 68% or 66% yield (76.6 mg or 247.8 mg), **C3** in a 65% or 62% yield (66.2 mg or 210.4 mg), and **C4** in a 72% or 67% yield (75.5 mg or 234.1 mg), respectively. The structures of all the target compounds were confirmed by IR, ^1^H NMR, and ^13^C NMR analysis (**Supplementary Materials**).

### Cytotoxic Activity *In Vitro* Studies

**Figure 2:**
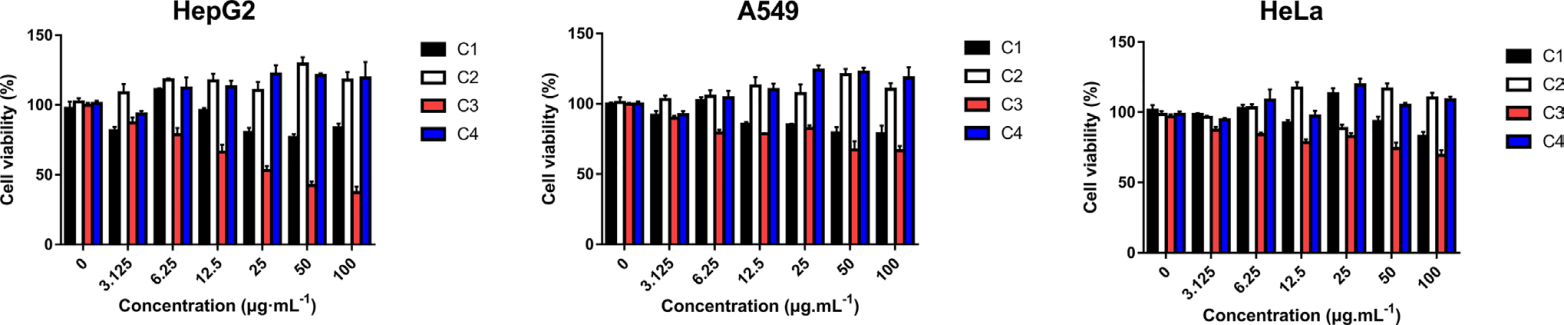
The anti-proliferative activities of indolizine derivatives on the cell lines (HepG2, A549, and HeLa) *via* MTT assay. Cells were exposed to different concentrations of indolizine derivatives **(C1, C2, C3,** and **C4)** for 24 h. The data were expressed as mean ± S.D. (*n* = 3).

Four new indolizine derivatives were tested for their cytotoxicity against the cell lines (HepG2, A549, and HeLa) by using the MTT assay. The cells’ viability was illustrated in **Figure 2**; it could be indicated that C3 could significantly inhibit the proliferation of the HepG2 cells. Thus, this cell line was used for further investigation regarding the underlying mechanisms accounting for the antiproliferative action of C3.

### C3 Suppresses the Proliferation of HepG2 Cells

**Figure 3:**
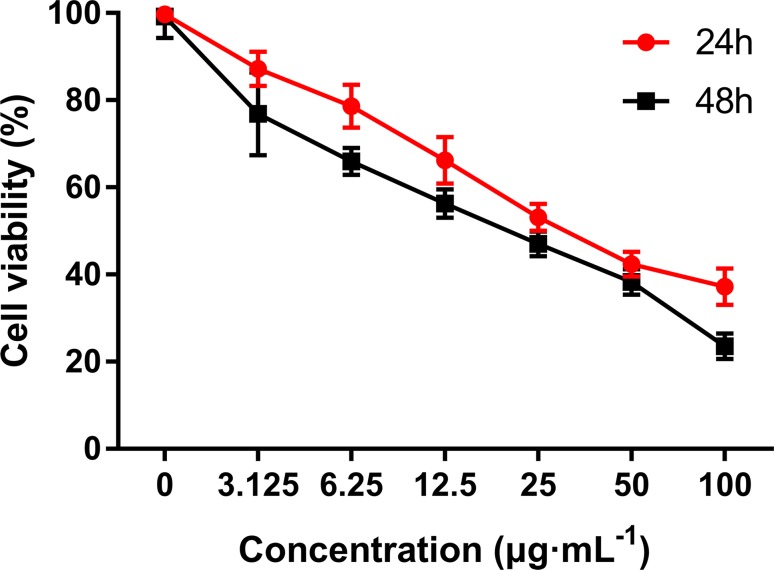
The anti-proliferative activities of C3 on HepG2 cells *via* MTT assay. Cells were exposed to different concentrations of C3 for 24 h and 48 h. The data were expressed as mean ± S.D. (*n* = 3).

MTT assay was performed to evaluate the growth and viability of HepG2 cells in different groups. As shown in **Figure 3**, the viability of HepG2 cells was significantly inhibited by C3 in a dose- and time-dependent manner. The IC_50_ values were calculated as 36.43 ± 0.69 and 19.41 ± 0.34 in cells treated for 24 and 48 h, respectively.

### Effects of C3 on Apoptosis in HepG2 Cells

**Figure 4:**
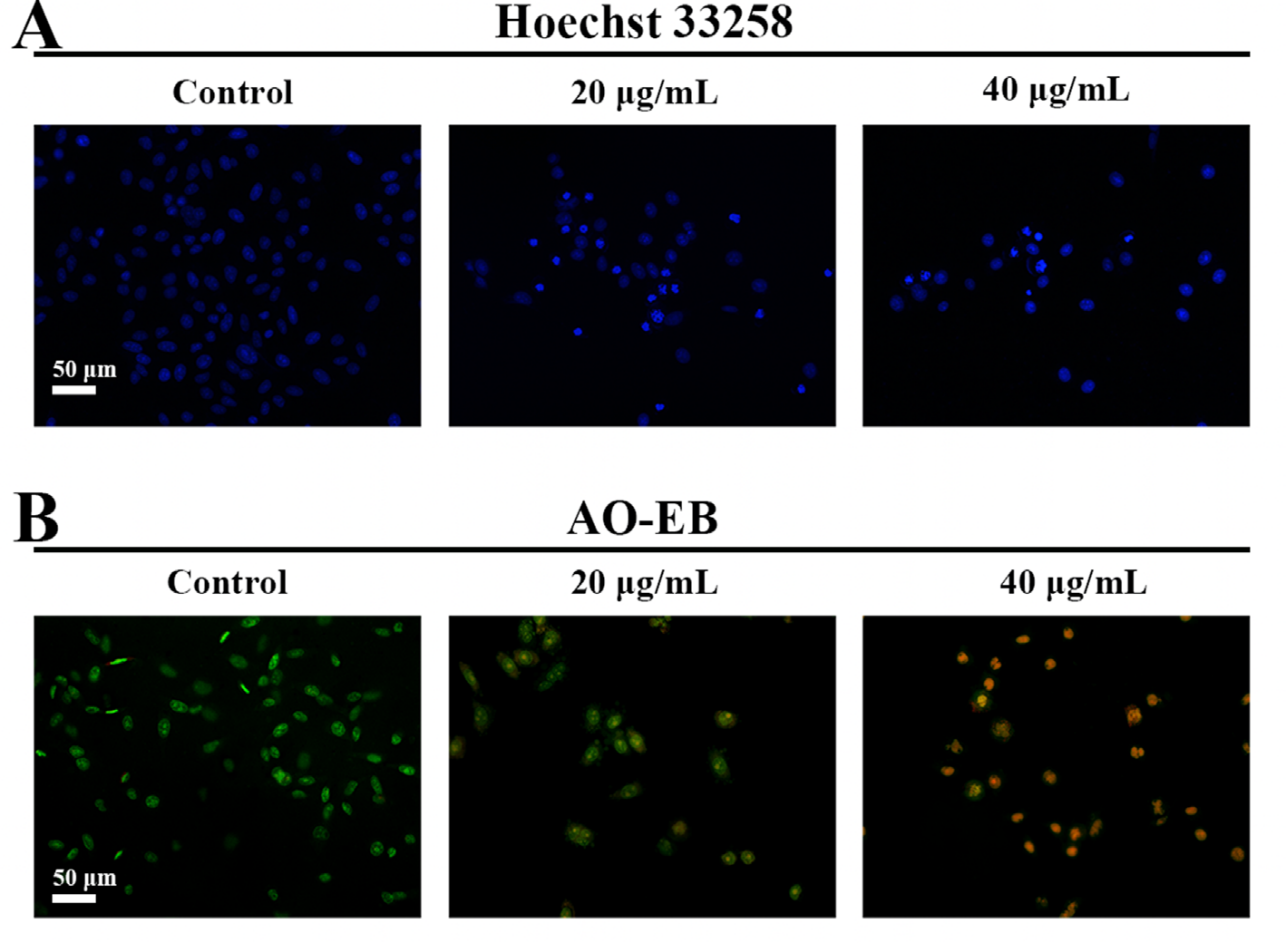
Morphological changes of HepG2 cell apoptosis were analyzed by fluorescent staining. **(A** and **B)** The cells were incubated with C3 (0, 20, and 40 µg/ml) for 24 h and stained by Hoechst 33258 and acridine orange/ethidium bromide (AO/EB) to observe the morphology. Scale bar = 50 µm.

**Figure 5:**
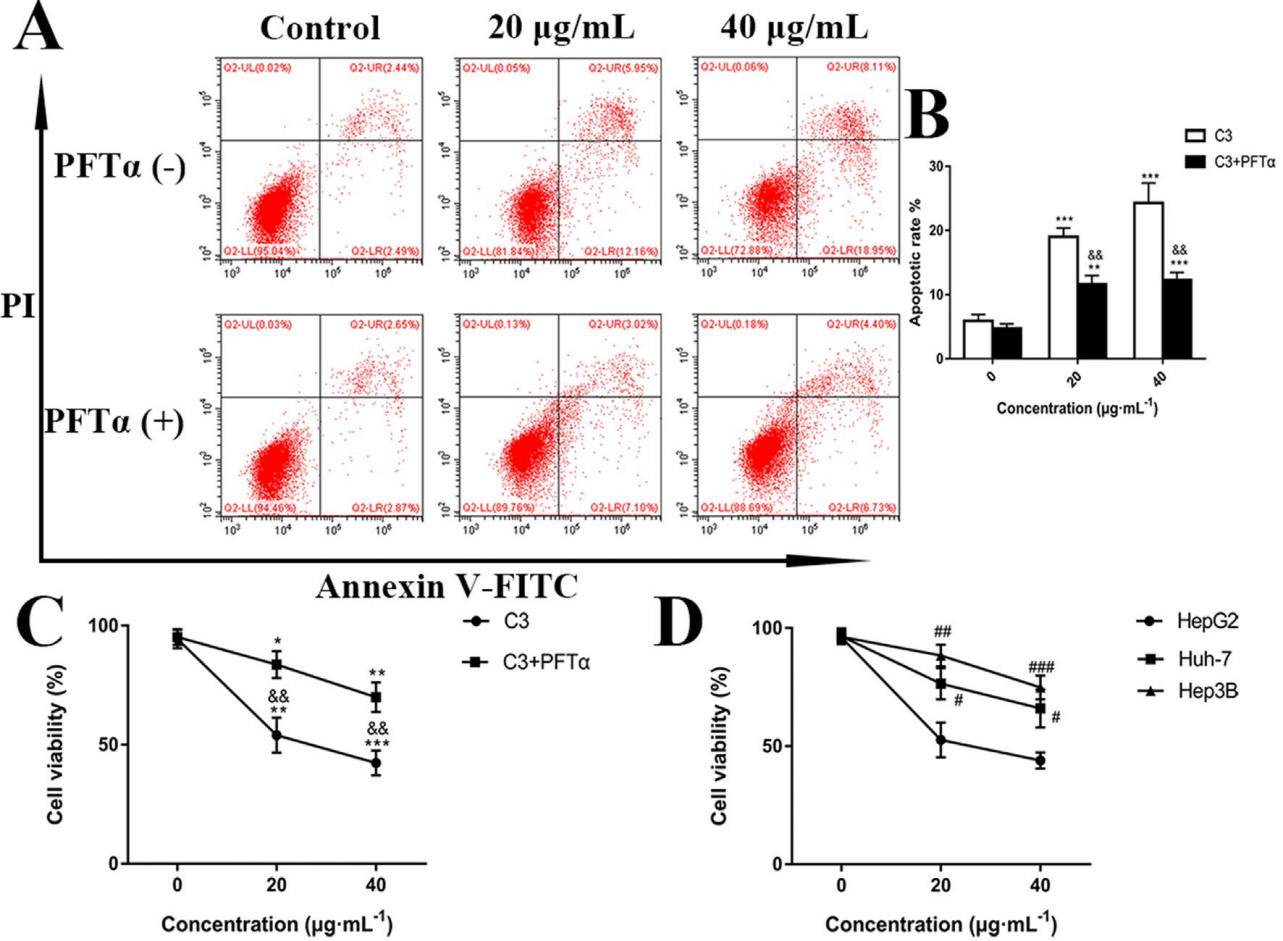
**(A)** Flow cytometry analysis of apoptosis in HepG2 cells. The cells were exposed to C3 and pifithrin-α (PFTα) (5 µM) co-treatment compared with C3 treatment alone for 24 h. **(B)** Quantitative analysis of the percentage of apoptotic cells in each treatment group. **(C)** In the absence or presence of PFTα (5 µM), the HepG2 cells’ viability was examined by MTT assay. **(D)** The cytotoxicity of C3 on three HCC cell lines with different p53 status (HepG2: p53-wild; Hep3B: p53-null; Huh-7: p53-mutant) was measured. The data were expressed as mean ± S.D. (*n* = 3). *P < 0.05, **P < 0.01, ***P < 0.001 compared to the control group; ^&&^ *P* < 0.01, compared to C3 treatment alone; ^#^ *P* < 0.05, ^##^ *P* < 0.01, ^###^ *P* < 0.001 compared to the HepG2 cells group.

Hoechst 33258 staining and AO/EB staining were used to observe the morphology of the cells with a fluorescence microscope. **Figure 4A** showed the captured images of the HepG2 cells by Hoechst 33258 fluorescence staining. In the control group, the nuclei of HepG2 cells were stained in blue and were uniform in shape. However, cell morphology altered significantly in C3 group. Condensed and bright chromatin were observed as well as some cell fragments that were morphological hallmarks of apoptotic cell death. This observation could be further certified in AO/EB staining. AO can stain both live and dead cells. EB is taken up only by cells that have lost their membrane integrity. Under the fluorescence microscope, viable cells show dark green color and apoptotic cells display yellow and orange color. (Nouri et al., 2018). It was clearly observed from **Figure 4B** that homogeneous green living cells with normal morphology were observed in the control group. The C3 group showed distinct yellow or orange fluorescence. All these typical apoptotic morphological changes suggested that C3 could induce HepG2 cell apoptosis. Meanwhile, the results of Annexin-V/PI double staining (**Figures 5A, B**) further suggested that as the concentration of C3 (20 and 40 μg/ml) increased, the ratios of cells in apoptosis significantly increased, while the percentage of viable cells decreased. The data above revealed that C3 induced apoptosis in HepG2 cells in a dose-dependent manner.

### Effects of C3 on MMP

**Figure 6:**
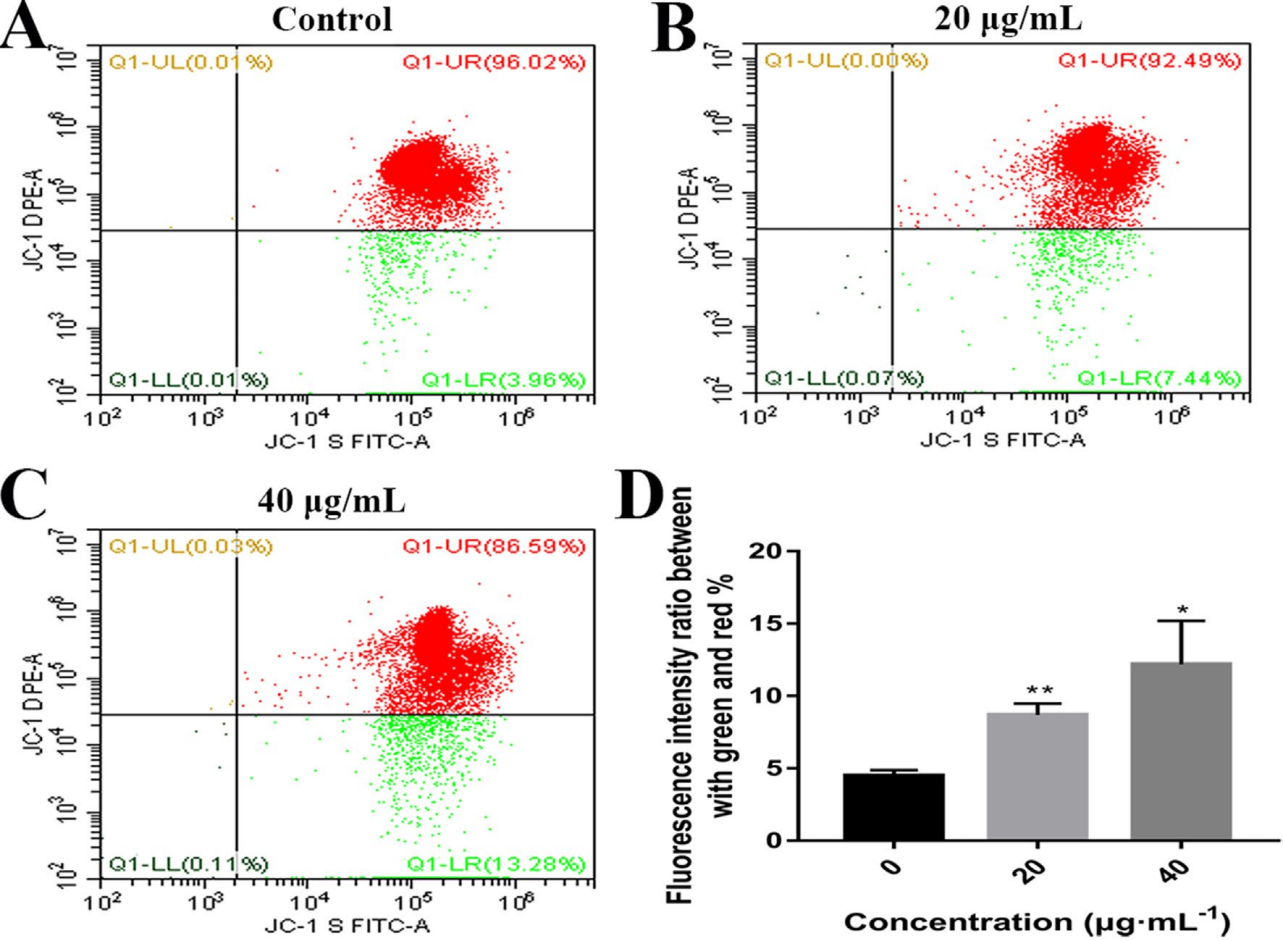
Effects of C3 on mitochondrial membrane potential (MMP). **(A–C)** HepG2 cells were incubated with C3 (0, 20, and 40 µg/ml) for 24 h and stained with JC-1 dye. **(D)** The ratio of green/red fluorescence was used to indicate MMP levels. The data were expressed as mean ± S.D. (*n* = 3). *P < 0.05, **P < 0.01 compared to the control group.

Mitochondrial alteration plays an essential role in cell apoptosis (Hengartner, 2000; Sheridan and Martin, 2010). To investigate the effects of C3 on mitochondrial function, flow cytometry was used to detect changes in MMP. As demonstrated in **Figure 6**, the untreated HepG2 cells exhibited abundance of red fluorescent aggregates with intact Δψm at 24 h. In contrast, C3 treatment (20 and 40 μg/ml) in HepG2 cells augmented the green fluorescent monomer population to 7.44% and 13.28% at 24 h, indicating that C3 could decrease the MMP. These results indicated that C3 might induce HepG2 cells apoptosis through the mitochondria-mediated apoptosis pathway.

### Effects of C3 on ROS Generation

**Figure 7:**
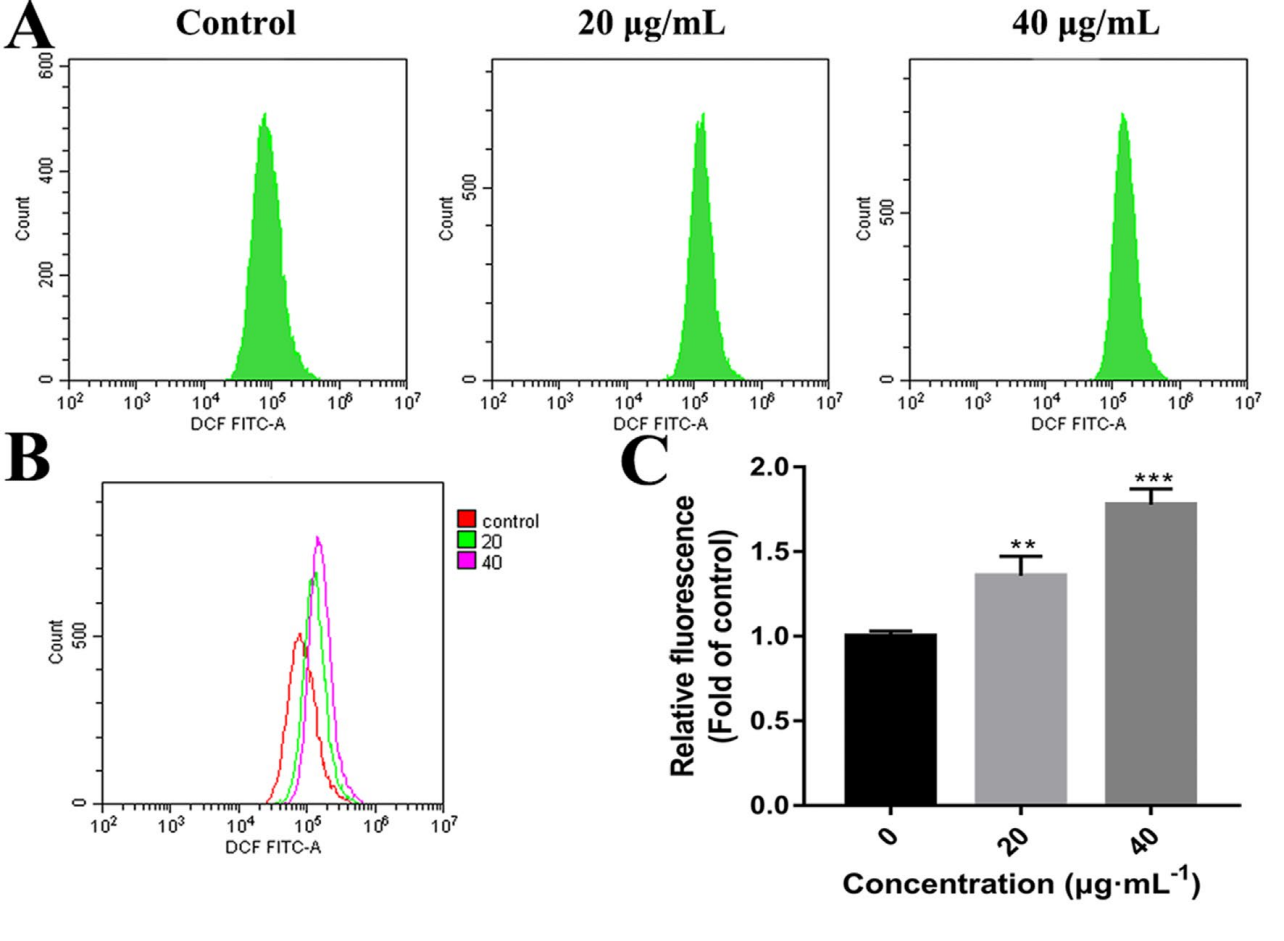
Reactive oxygen species (ROS) generation in HepG2 cells incubated with C3. **(A, B)** After incubated with C3 (0, 20, and 40 µg/ml) for 24 h, the levels of intracellular ROS were detected by DCFH-DA (2′,7′-Dichlorodihydrofluorescein diacetate) assay. **(C)** The levels of ROS were presented as fold change compared to the levels in the control group. The data were expressed as mean ± S.D. (*n* = 3). **P < 0.01, ***P < 0.001 compared to the control group.

High concentrations of ROS can destroy mitochondrial membranes and reduce MMP, which activates the mitochondrial pathway of apoptosis (Chong et al., 2014; Idelchik et al., 2017). To examine whether C3 promoted the generation of ROS in HepG2 cells, ROS levels were assessed by detecting DCF fluorescence intensity using flow cytometry. The elevation in the intracellular ROS level by C3 (20 and 40 μg/ml) was evident by the right shift of the histogram (**Figures 7A, B**). C3 dose-dependently (20 and 40 μg/ml) enhanced the production of intracellular ROS level by 1.36 ± 0.12-folds and 1.78 ± 0.09 folds, respectively, compared to the untreated cells (**Figure 7C**).

### C3 Treatment Induced p53 Expression and Nuclei Accumulation in HepG2 Cells

**Figure 8:**
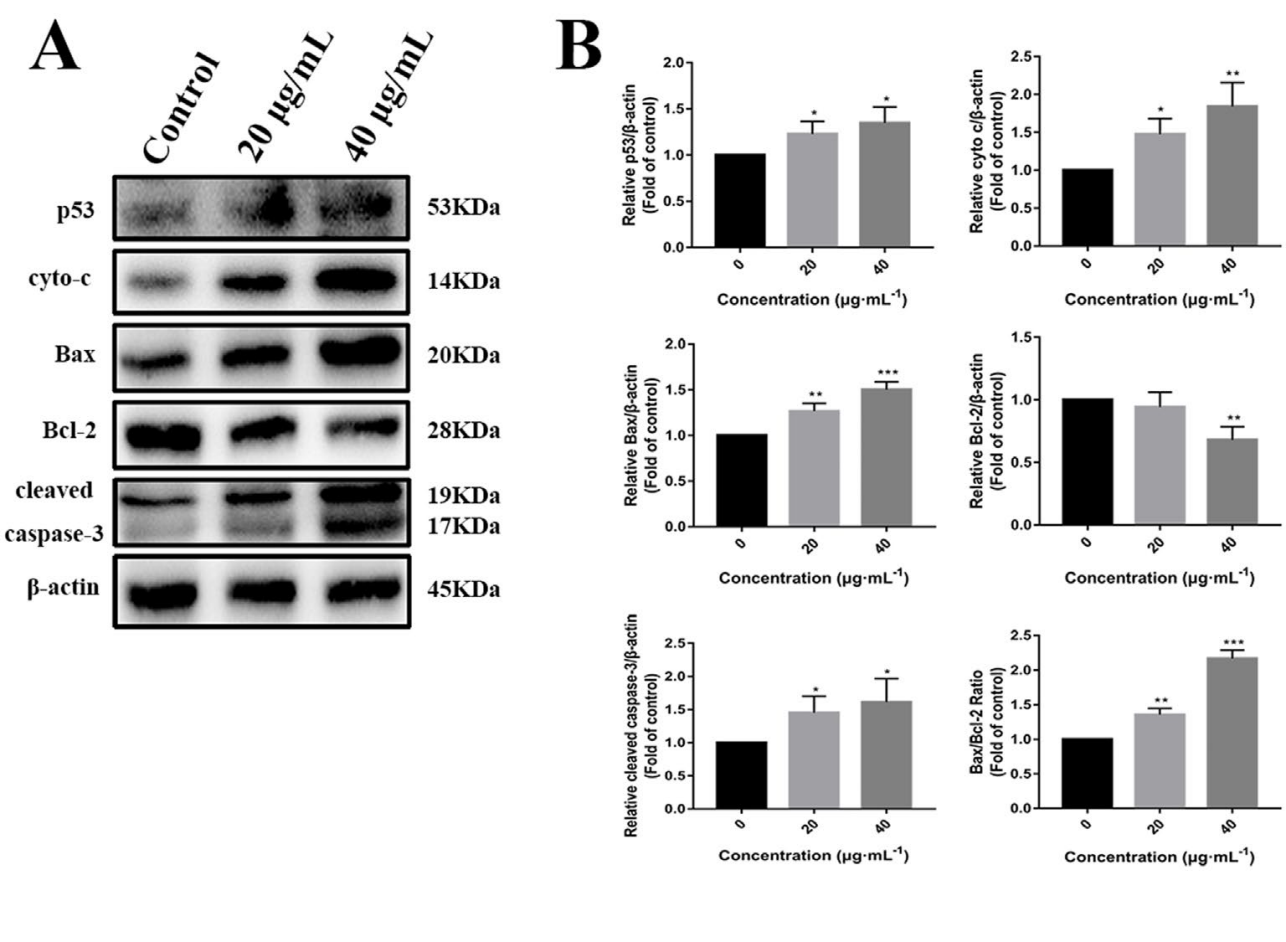
**(A)** Western blot analysis of p53, cyto-c, Bax, Bcl-2, and cleaved caspase-3 in HepG2 cells incubated with C3 (0, 20, and 40 µg/ml) for 24 h. β-actin was used as the internal control. **(B)** The quantified levels of each protein were analyzed by Image J soft. The data were expressed as mean ± S.D. (*n* = 3). *P < 0.05, **P < 0.01, ***P < 0.001 compared to the control group.

**Figure 9:**
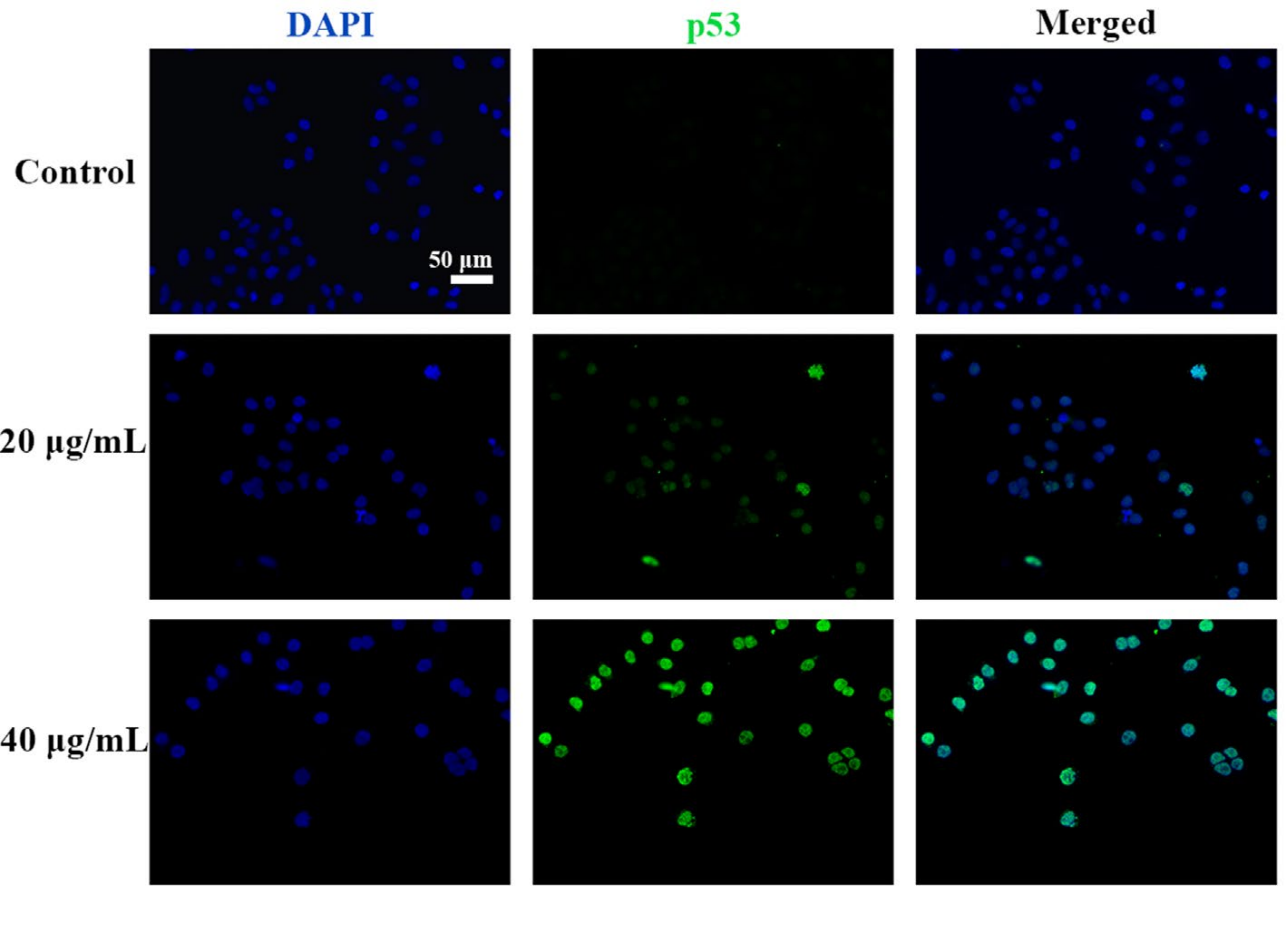
C3 activated nuclei p53. HepG2 cells incubated with C3 (0, 20, and 40 µg/ml) for 24 h and was subjected to immunofluorescence staining. Green is for p53, blue is for nucleus. Scale bar = 50 µm.

p53 is a key tumor suppressor protein that plays a central role in the process of apoptosis by integrating various signaling pathways (Labuschagne et al., 2018). As shown in **Figure 8**, in the C3 group, the expression of p53 increased obviously in a dose-dependent manner after 24 h. Then, p53 localization in HepG2 cells was determined by means of immunofluorescence staining with fluorescence microscopy. The results showed that C3 stimulated p53 accumulation in nuclei (**Figure 9**). These findings indicated that C3 could promote p53 activation. To further confirm the functional involvement of p53 in the C3-induced proliferation inhibition on HepG2 cells (p53-wild), two other HCC cell lines with different p53 status (Hep3B: p53-null; Huh-7: p53-mutant) were used for the evaluation of cytotoxicity. We also investigated whether PFTα, a specific inhibitor of p53, affects C3-mediated apoptotic cell death to further demonstrate the pivotal role of p53 in reduced HepG2 cell viability following C3 treatment. The results indicated that the proliferation inhibition of C3 was more sensitive to HepG2 cells compared to the other two HCC cells (**Figure 5D**). Compared with the C3-treatment-alone group, higher HepG2 cell viability and lower apoptotic rate preferred to appear in the 5 µM PFTα co-treatment group (**Figures 5A–C**). When taken together, these results implied the involvement of p53 in C3-triggered cell death in HepG2 cells.

### The Mitochondrial p53 Pathway Is Involved in C3-Induced Apoptosis in HepG2 Cells

To further investigate the role of the mitochondrial p53 pathway in C3-induced apoptosis of HepG2 cells, some related proteins were assessed by Western blot analysis, including Bax, Bcl-2, cytochrome c, and cleaved caspase-3. β-actin was used as the internal control. The C3 group significantly increased the Bax protein and decreased the Bcl-2 protein, causing the ratios of Bax/Bcl-2 to increase markedly. It was found that the expression of cleaved caspase-3 and cytochrome c was increased in the cells incubated with C3 (**Figure 8**). Taken together, all these results suggested that the mitochondrial p53 pathway is involved in the apoptosis induced by C3.

## Discussion

In this study, four novel indolizine derivatives were synthesized. C3 induced apoptosis in HepG2 cells and possible molecular mechanisms were first evaluated. The results demonstrated that C3 could induce apoptosis in HepG2 cells *via* the mitochondrial p53 pathway.

Apoptosis is a form of programmed cell death that is characterized by a series of morphological changes including cell shrinkage, chromatin condensation, and formation of apoptotic bodies (Kerr et al., 1972). In our fluorescence staining experiments, significant morphological changes were observed in C3-treated HepG2 cells. Furthermore, the results of Annexin-V/PI double staining exhibited an obvious increase in the proportion of early and late apoptosis. Caspase-3, a member of the cysteine-aspartic acid protease (caspase) family, acts as a major executioner of apoptotic death. Once caspase-3 is activated, it cleaves key cellular proteins and dismantles the cell (Koff et al., 2015; Larsen and Sørensen, 2017). C3 stimulated activation of caspase-3 (**Figure 8**), which may be one of the vital reasons for triggering the antiproliferative activity of C3 on HepG2 cells.

There are two known signaling pathways mediating apoptosis: the extrinsic and intrinsic pathways. The extrinsic pathway is mediated by cell surface death receptors, while the intrinsic pathway is initiated in the mitochondria (Khan et al., 2014). Activation of the mitochondrial apoptotic pathway includes decrease of mitochondrial transmembrane potential and increase of ROS (Wang et al., 2012; Wang et al., 2016). This study implied that C3 promoted the generation of ROS and decreased the MMP (**Figures 6** and **7**). Increase of ROS can cause MMP loss by activating mitochondrial permeability transition that in turn activates the Bcl-2 family proteins and promotes cytochrome c release, leading to apoptosis (Xiao et al., 2009; Moloney and Cotter, 2018). The Bcl-2 protein family includes a variety of anti-apoptotic proteins such as Bcl-2 and pro-apoptotic proteins such as Bax. The ratio of Bax/Bcl-2 is the key to the permeabilization of the mitochondrial outer membrane and regulating apoptosis (Juin et al., 2013; Gross, 2016; Garner et al., 2017). Results of Western blot (**Figure 8**) indicated that the expression of the anti-apoptotic protein Bcl-2 was inhibited, while the expression of the pro-apoptotic protein Bax was activated compared with the control group. The proportion of Bax/Bcl-2 increased significantly. Furthermore, the release of cytochrome c also increased obviously compared with the control group. These results further confirmed that C3 promoted mitochondria-mediated apoptosis. Therefore, it is worth noting that C3 induced mitochondria-mediated apoptosis in HepG2 cells by increasing the Bax/Bcl-2 ratio, promoting cytochrome c release, and activating caspase-3.

The p53, a tumor suppressor, is known to induce apoptosis and plays an important role in mitochondria-mediated apoptosis (Wickramasekera and Das, 2014; Wang, 2015). In our study, we investigated the role of the tumor suppressor p53 in C3-induced apoptosis using cells with different genotypic profiles, including wild-type p53 HepG2, p53-null Hep3B, and mutant p53 Huh-7 (Muller et al., 1997). It was found that C3 markedly depressed the proliferation of HepG2 cells compared with other cell lines (**Figure 5D**). According to some reports, activation of p53 is associated with the increase of ROS production (Yin et al., 2015; Chen et al., 2017). As shown in **Figure 9**, accumulation of p53 in nuclei was observed in the C3 group. It is known that activated p53 accumulates in the nuclei and activates apoptosis (Li et al., 2012; Zhou et al., 2015; Tu et al., 2018). To define the role of p53 activation in C3-induced apoptosis, PFTα, a p53 specific inhibitor, was applied to attenuate p53 activity. The results indicated that the HepG2 cell viability of the 5 µM PFTα co-treatment group increased compared with the C3 treatment group alone. Similarly, the cells’ apoptosis rate decreased by 7.99% (20 µg/ml group) and 15.93% (40 µg/ml group) with PFTα co-treatment, compared to the rate with C3 treatment alone **(** **Figures 5A–C**). These results indicated that activation of p53 was involved in C3-induced apoptosis of HepG2 cells. Furthermore, the increased expression of the p53 protein with C3 treatment also verified the above results. It has been documented that p53 regulates the expression of different members of the Bcl-2 family proteins such as the anti-apoptotic Bcl-2 protein and the pro-apoptotic Bax protein, thus promoting mitochondria-mediated apoptosis (Dashzeveg and Yoshida, 2015; Kruiswijk et al., 2015). The activated p53 can promote depolarization of Δψm by forming complexes with pro-apoptotic Bcl-2 family proteins. This study suggested that the p53 activation then leads to the up-regulation of Bax and the down-regulation of Bcl-2. It was the imbalance of Bax/Bcl-2 that leads to mitochondrial dysfunction, which induced mitochondria-mediated apoptosis.

A novel indolizine derivative C3 in this study was provided with significant inhibition of proliferation in HepG2 cells, and the new mechanisms were first discovered. The p53 accumulation in nuclei was stimulated and ROS production was promoted in the cells with C3 treatment, resulting in up-regulated expression of Bax and down-regulated expression of Bcl-2. These led to MMP depolarization and cytochrome c release, which ultimately induced caspase-3 activation. The findings revealed that C3 induced apoptosis through the mitochondrial p53 pathway in HepG2 cells. C3 is expected as a potential candidate for liver cancer.

## Data Availability

All datasets generated or analyzed for this study are included in the manuscript and the supplementary files.

## Author Contributions

YL, ES, and ZZ performed research. YL and DY contributed new reagents/analytic tools. ES analyzed data. GL participated in some experiments. YL wrote the paper. HH and HC contributed to the study design and helped in the review and editing of the original article.

## Funding

This work was supported by grants from the Special Funds of the Central Finance to Support the Development of Local Universities and Colleges, and Guangdong Province Department of Education (grant 2015KGJHZ022). It was supported also by the projects of Guangzhou key laboratory of construction and application of new drug screening model systems (no. 201805010006) and Key Laboratory of New Drug Discovery and Evaluation of ordinary universities of Guangdong province (no. 2017KSYS002).

## Conflict of Interest Statement

The authors declare that the research was conducted in the absence of any commercial or financial relationships that could be construed as a potential conflict of interest.
