# Supplementary figures and images for: A Novel Indolizine Derivative Induces Apoptosis Through the Mitochondria p53 Pathway in HepG2 Cells
Source: Front Pharmacol. 2019 Jul 10;10:762. doi: 10.3389/fphar.2019.00762 (PMC6635656; doi:10.3389/fphar.2019.00762)

**C1: 1H NMR**


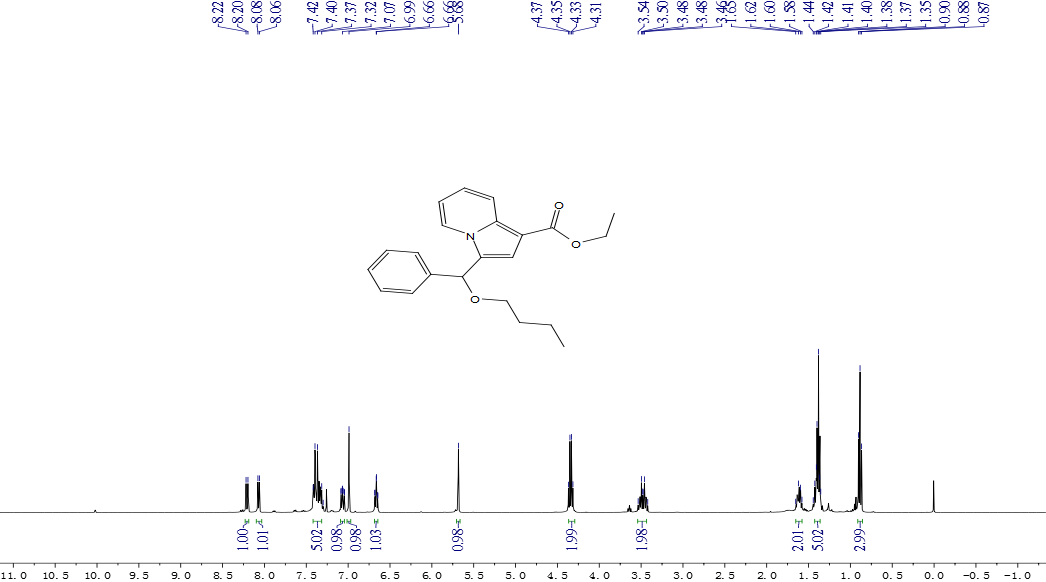


**C1: 13C NMR**


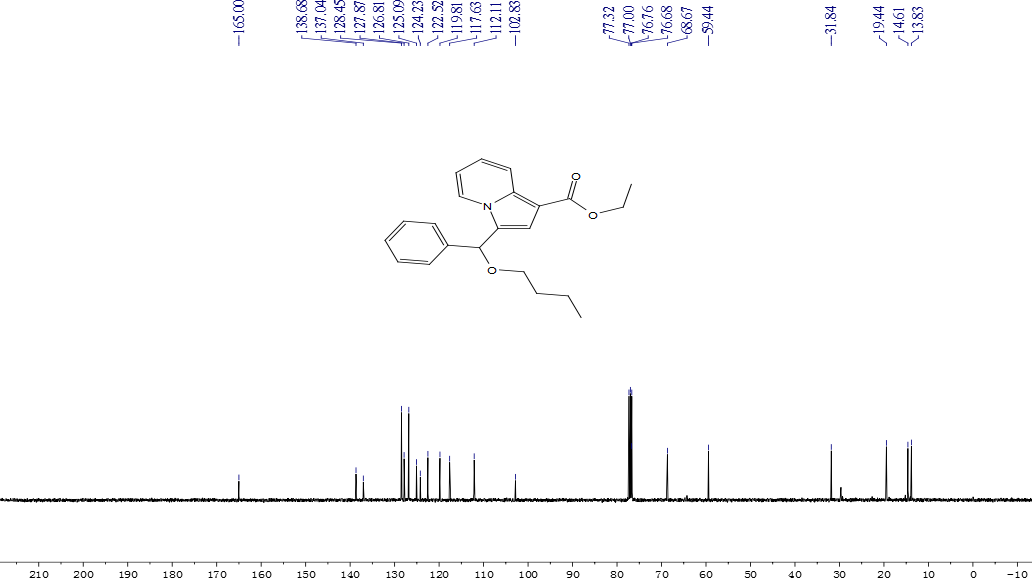


**C1: IR**

**
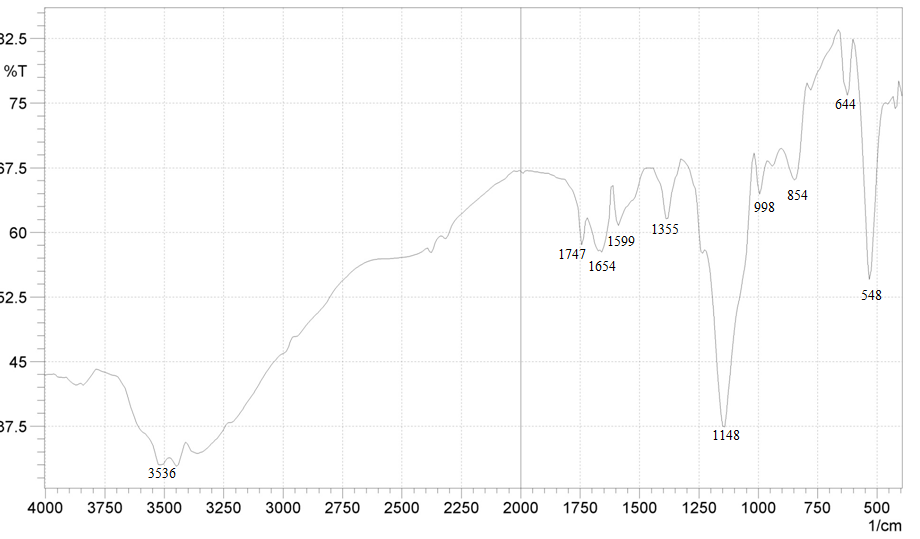
**

**C2: 1H NMR**


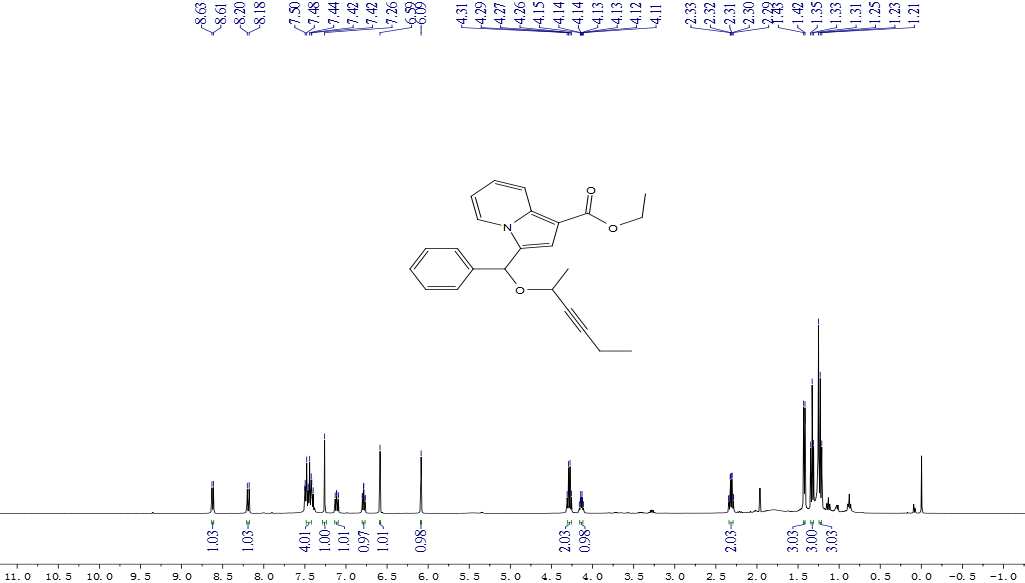


**C2: 13C NMR**


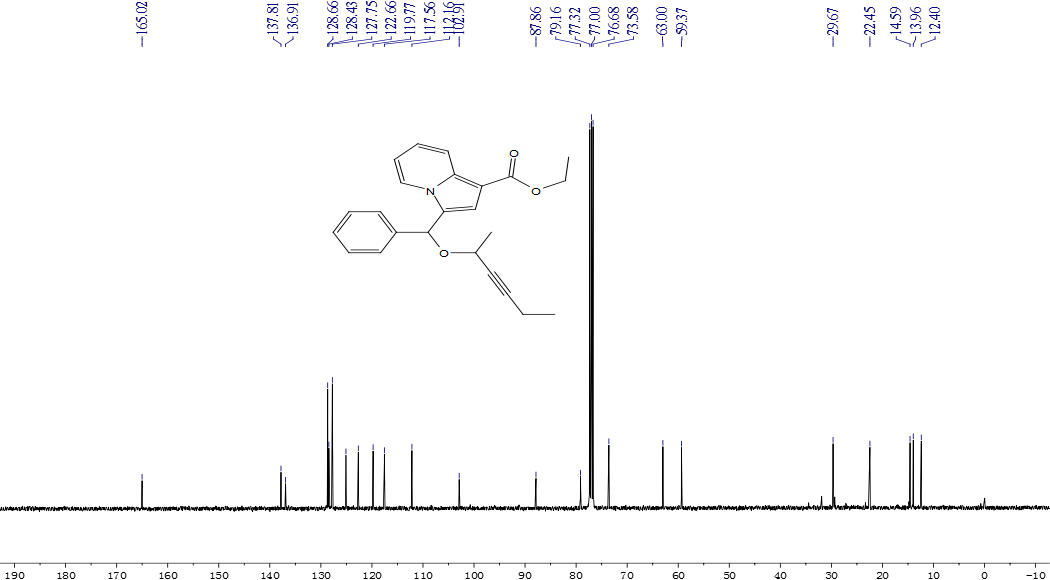


**C2: IR**

**
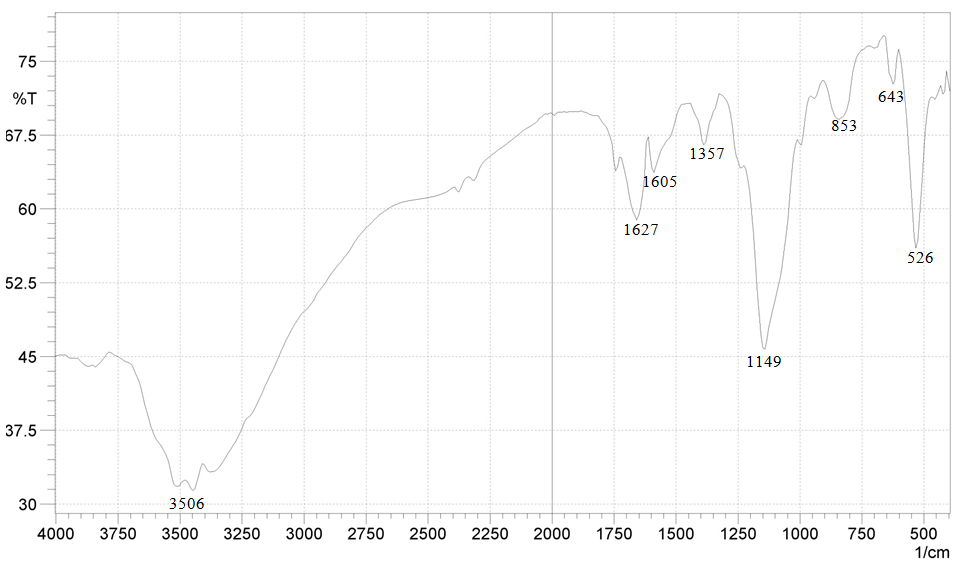
**

**C3: 1H NMR**


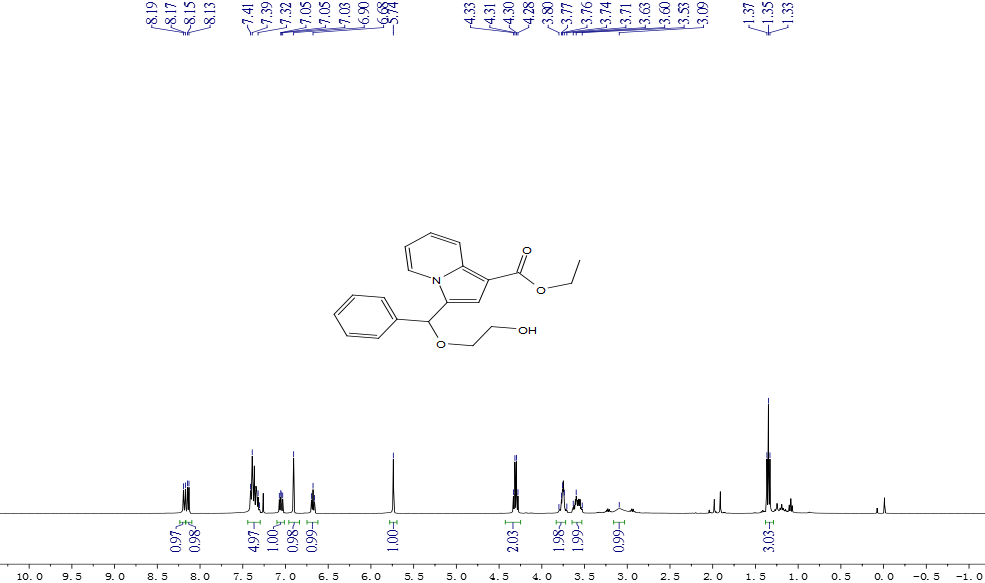


**C3: 13C NMR**


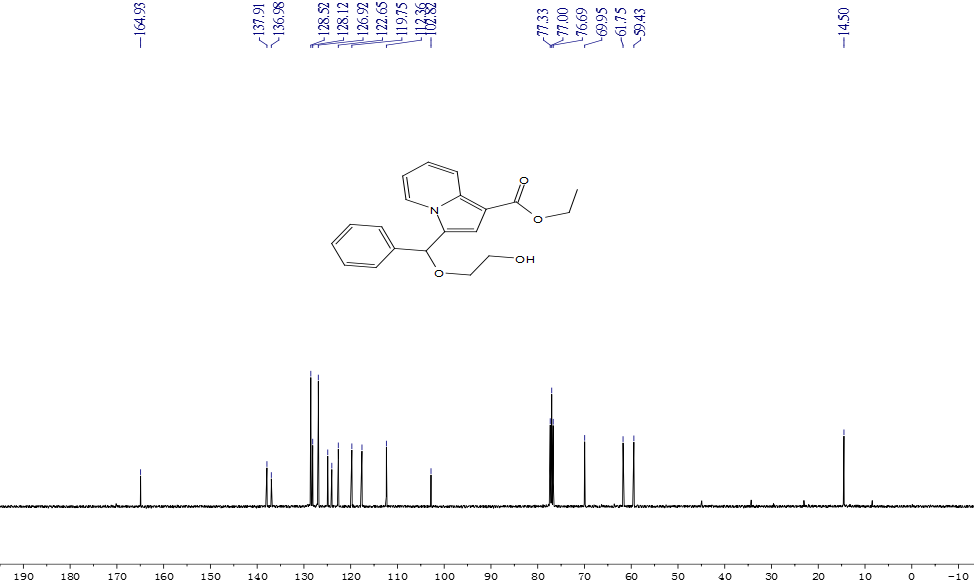


**C3: IR**

**
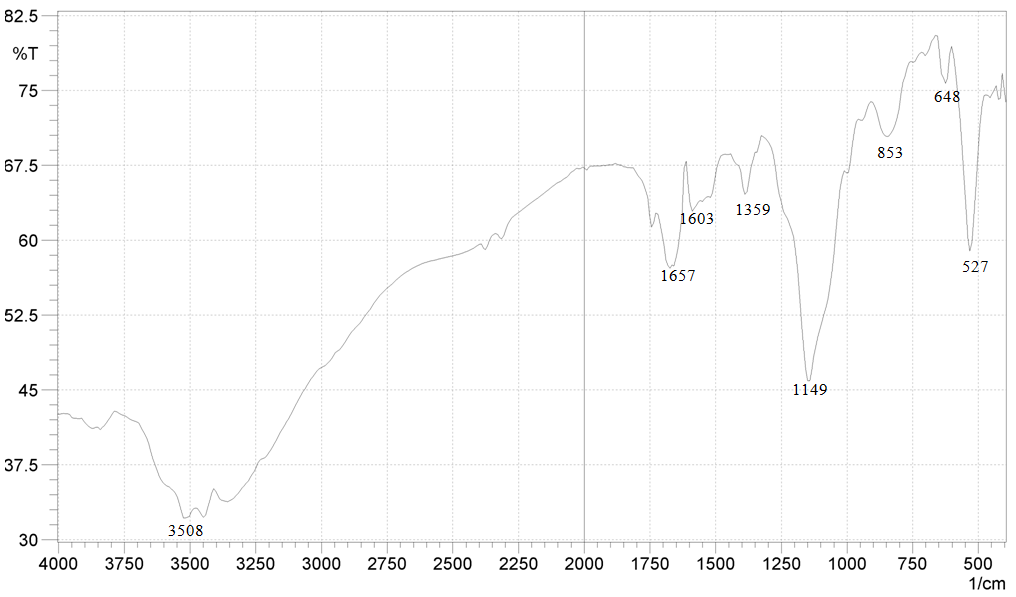
**

**C4: 1H NMR**


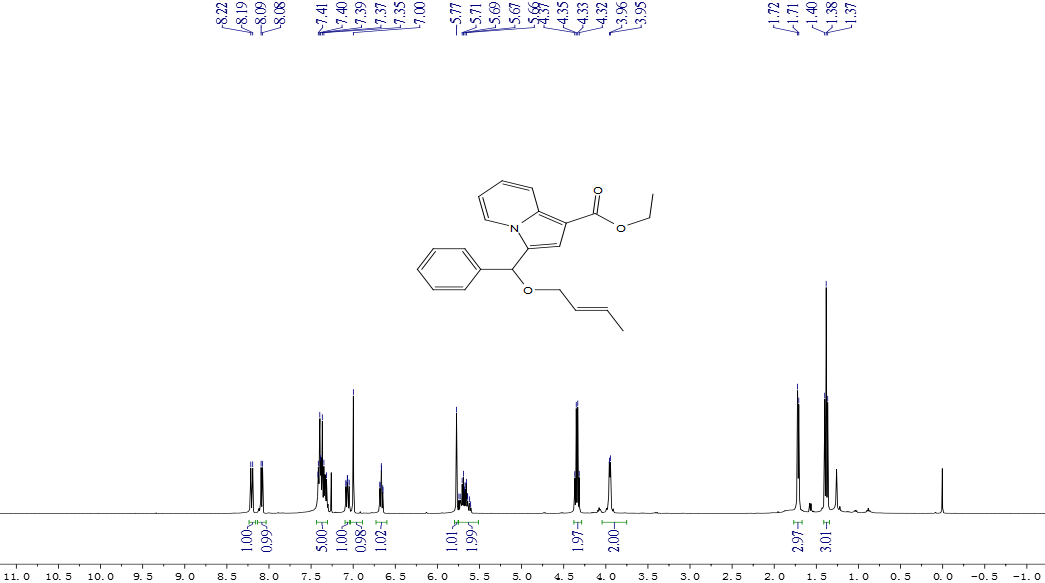


**C4: 13C NMR**


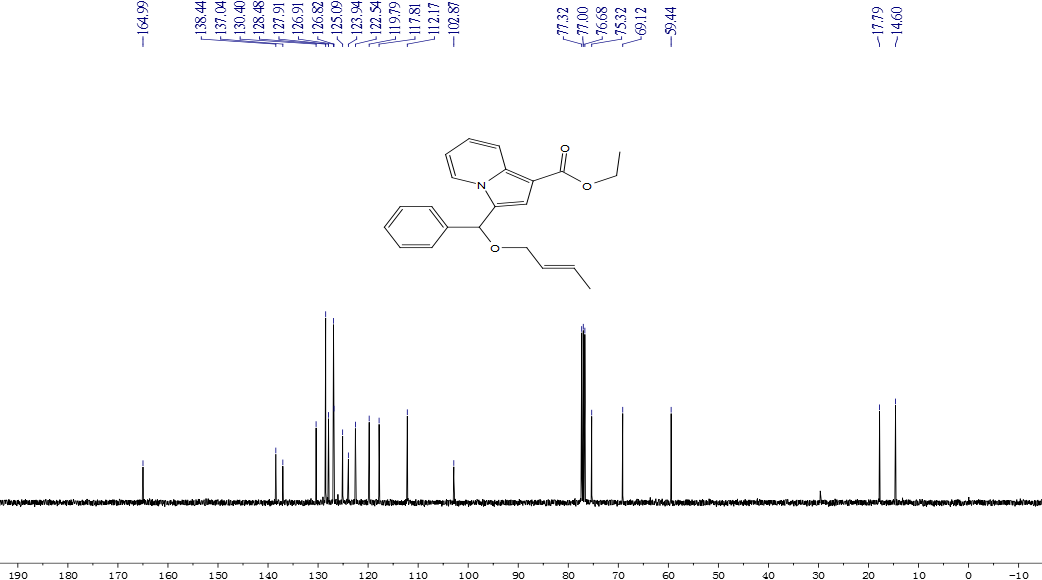


**C4: IR**

**
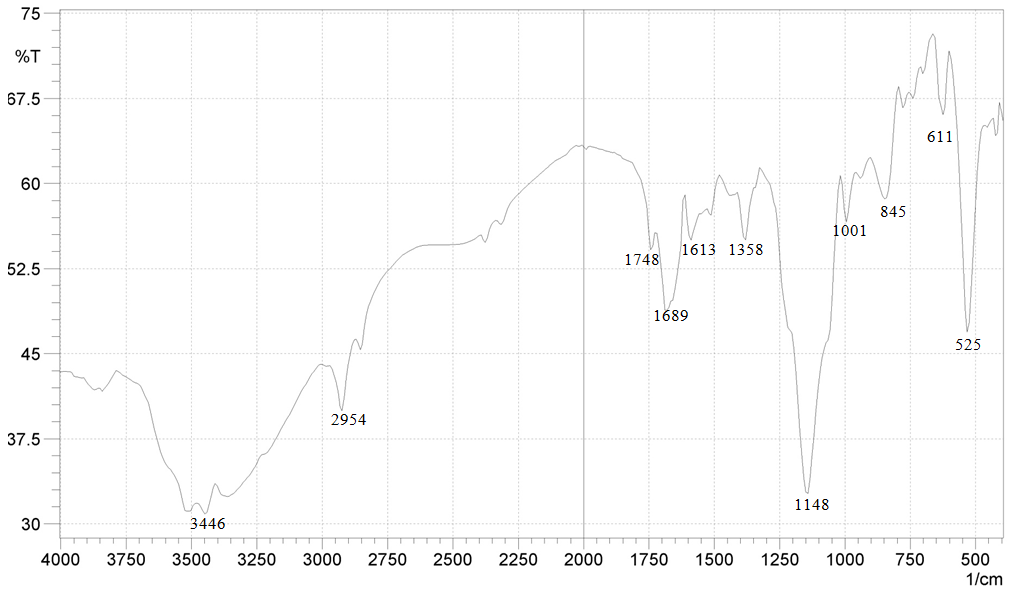
**

Supplement: Supplementary file 1 [file Table_1.docx]
